# Rational Uniform Consensus with General Omission Failures

**DOI:** 10.1155/2022/9544059

**Published:** 2022-08-31

**Authors:** Yansong Zhang, Bo Shen, Yingsi Zhao

**Affiliations:** ^1^School of Electronic and Information Engineering, Beijing Jiaotong University, Beijing, China; ^2^Key Laboratory of Communication and Information Systems, Beijing Municipal Commission of Education, Beijing, China; ^3^School of Economics and Management, Beijing Jiaotong University, Beijing, China

## Abstract

Generally, system failures, such as crash failures, Byzantine failures, and so on, are considered as common reasons for the inconsistencies of distributed consensus and have been extensively studied. In fact, strategic manipulations by rational agents are not ignored for reaching consensus in a distributed system. In this paper, we extend the game-theoretic analysis of consensus and design an algorithm of rational uniform consensus with general omission failures under the assumption that processes are controlled by rational agents and prefer consensus. Different from crashing one, agent with omission failures may crash or omit to send or receive messages when it should, which leads to difficulty of detecting faulty agents. By combining the possible failures of agents at the both ends of a link, we convert omission failure model into link state model to make faulty detection possible. Through analyzing message passing mechanism in the distributed system with *n* agents, among which *t* agents may commit omission failures, we provide the upper bound on message passing time for reaching consensus on a state among nonfaulty agents and message chain mechanism for validating messages. Then, we prove that our rational uniform consensus is a Nash equilibrium when *n* > 2*t* + 1, and failure patterns and initial preferences are blind (an assumption of randomness). Thus, agents have no motivation to deviate the consensus, which could provide interpretable stability for the algorithm in multiagent systems such as distributed energy systems. Our research strengthens the reliability of consensus with omission failures from the perspective of game theory.

## 1. Introduction

How to reach consensus despite failures is a fundamental problem in distributed computing. In consensus, each process proposes an initial value and then executes a unique consensus algorithm independently. Eventually all processes need to agree on a same decision chosen from the set of initial values even if there may be some system failures, such as crash failures, omission failures, and Byzantine failures [1]. In the crash model, processes can get into failure state by stopping executing the remaining protocol. In the omission model, processes can get into failure state by omitting to send or receive messages. Also, in the Byzantine model, processes can fail by exhibiting arbitrary behavior. Extensive studies have been conducted on fault-tolerant consensus.

Moreover, two kinds of consensus problems are usually distinguished. One is non-uniform version (usually called “consensus” directly) where no two nonfaulty processes decide differently. The other is uniform version (called “uniform consensus”) where no two processes (whether correct or not) decide on different values. We believe that consensus protocols cannot simply replace uniform consensus protocols because the condition of non-uniform consensus is inadequate for many applications [2]. From [3], uniform consensus is harder than consensus because one additional round is needed to decide. Also, uniform consensus is meaningless with Byzantine failures.

Game theory provides interpretable equilibrium by analyzing the game among intelligent players. We argue that its incentive mechanism and punishment mechanism can be effectively applied in distributed systems. Recently, there is an increasing interest on distributed game theory especially in several fields such as peer-to-peer network, biological system, cryptocurrency, and e-commerce, in which processes are selfish called rational agents (or intelligent agents). Combining distributed computing with algorithmic game theory is an interesting research area enriching the theory of fault-tolerant distributed computing. In this framework, agents may deviate from protocols with any behaviors in order to increase their own profits according to utility functions, which could be regarded as general Artificial Intelligence. In [4], this kind of deviation is referred to as strategic manipulation of distributed protocol. This research is necessary in some practical scenarios, in which each process has selfish incentives. Also, we argue that the fairness of algorithms must be promoted by game theory. Clearly, the goal of distributed computing in the context of game theory is to design algorithms for reaching Nash equilibrium, in which all agents have no incentive to deviate from the algorithms. Perhaps, this framework has been investigated and formalized for the first time in the context of secret sharing and multiparty computation [5–8]. More recently, some fundamental tasks in distributed computing such as leader election and consensus have been studied from the perspective of game theory [9–17].

Following this new line of research, we combine fault-tolerant consensus with rational agents and study the rational uniform consensus problem in synchronous round-based system, where every agent has its own preference on consensus decisions. Thus, an algorithm of rational uniform consensus needs to be constructed. Also, for each agent, its utility is not less with following the consensus algorithm than with deviating from the algorithm. That achieves a Nash equilibrium. It is easy to see that standard consensus algorithms cannot reach equilibrium and they can be easily manipulated by even a single rational agent. Several research studies on rational consensus have been conducted [4, 12–16, 18, 19], but none of them consider the uniform property. Also, most studies on rational consensus only support that there are crash failures or no system failures. We argue that omission failures, which are more subtle and complicated than crashing one, cannot be ignored for reaching uniform consensus. In this paper, we pay attention to a distributed system with *n* agents, among which *t* agents may experience omission failures. In this setting, we extend the game-theoretic analysis of consensus. Specifically, our contributions in this paper include the following:

- We utilize a punishment mechanism to convert omission failure model into link state model, which makes faulty detection more direct. In the link state model, faulty links never recover whether or not omission failures recover. Therefore, it can provide an idea to simplify the problem of faulty recovery in distributed computing.
- An almost complete mechanism analysis is given for message passing in the distributed system with general omission failures. Then, we provide the upper bound *x*+1 on message passing time for reaching consensus on a link state. The upper bound determines the round complexity of our algorithm. Next, a message chain mechanism is introduced for validating messages.
- An algorithm of rational uniform consensus with agent omission failures is presented for any *n* > 2*t*+1. We give a complete formal proof of correctness of our algorithm. The proof shows that our consensus is a Nash equilibrium.

The rest of the paper is organized as follows.  Section 2 introduces the related work.  Section 3 describes the model that we are working on.  Section 4 presents the algorithm of rational uniform consensus for achieving Nash equilibrium and proves it correct.  Section 5 concludes the paper.

## 2. Related Work

From the view point of modeling methods about agents, the research framework for distributed game theory in the literature may be divided into three categories. In the first category, all of the agents in distributed system are controlled by rational agents preferring consensus and some of them may randomly fail by system failures. Bei et al. [4] studied distributed consensus tolerating both unexpected crash failures and strategic manipulations by rational agents. They considered agents that may fail by crashing. However, the correctness of their protocols needs a strong requirement that it must achieve agreement even if agents deviate. Afek et al. [18] proposed two basic rational building blocks for distributed system and presented several fundamental distributed algorithms by using these building blocks. However, their protocol is not robust against even crash failures. Halpern and Vilaça [12] presented a rational fair consensus with rational agents and crash failures. They used failure pattern to describe the random crash failures of agents. Clementi et al. [13] studied the problem of rational consensus with crash failures in the synchronous gossip communication model. The protocols of Halpern et al. and Clementi et al. do not tolerate omission failure, but we think the consideration to it is necessary. Harel et al. [15] studied the equilibria of consensus resilient to coalitions of *n* − 1 and *n* − 2 agents. They gave a separation between binary and multi-valued consensus. However, they assumed that there are no faulty agents.

The second category is named rational adversary. Groce et al. [19] studied the problem of Byzantine agreement with a rational adversary. Rather than the first model, they assumed that there are two kinds of processes: one is honest and follows the protocol without question; the other is a rational adversary and prefers disagreement. Amoussou-Guenou et al. [14] studied Byzantine fault-tolerant consensus from the game theory point. They modeled processes as rational players or Byzantine players and consensus as a committee coordination game. In [14], the Byzantine players have utility functions and strategies, which can be regarded as rational adversaries similar to [19]. In our opinion, this framework limits the scope of the Byzantine problem.

Finally, the BAR framework (Byzantine, Altruistic, and Rational) was proposed in [20]. In [16], Ranchal-Pedrosa and Gramoli studied the gap between rational agreements that are robust against Byzantine failures and rational agreements that are robust against crash failures. Their model consists of four different types of players: correct, rational, crash, or Byzantine, which is similar to the BAR model. They consider that rational players prefer to cause a disagreement than to satisfy agreement, which we view as a bit limited because only referring rational players as rational adversaries is one of the questions in the Byzantine model. Moreover, no protocols are proposed in [16].

## 3. Model

We consider a synchronous system with *n* agents and each of agent has a unique and commonly known identify in *N*={1,…, *n*}. Execution time is divided into a sequence of rounds. Each round is identified by the consecutive integer starting from 1. There are three successive phases in a round: a send phase in which each agent sends messages to other agents in system, a receive phase in which each agent receives messages that are sent by other agents in the send phase of the same round, and a computation phase where each agent verifies and updates the value of local variables and executes local computation based on the messages sent and received in that round. We assume that every pair of agents *i* and *j* in *N* is connected by a reliable communication link denoted by link_*ij*_. For an agent *i*, all links in the system can be divided into two types: direct link link_*ij*_ where *j* ∈ *N*, and indirect link link_*kp*_ where neither *k* nor *p* is equal to *i*.

### 3.1. Failure Model

Here the general omission failures [21], which occur in agents and not in communication links [22], are considered. That is, an agent crashes or experiences either send omissions or receive omissions. Also, send omission means that the agent omits sending messages that it is supposed to send. Receive omission means that the agent omits receiving messages that it should receive. We define that agent omission failures never recover. We argue that our protocol also works even if failures could recover, but proving this seems more complicated. It is easy to see that crash failure can be converted to omission failure because if an agent crashes, it must omit to send and receive messages with all other agents after it has crashed. We assume that there are *t* agents undergoing general omission failures.

Based on the failure model, we divide the agents in the system into three types:

- *Good Agent*. Good agents do not have omission failures.
- *Risk Agent*. Risk agents experience omission failures but we temporarily consider them as correct agents in our protocol.
- *Faulty Agent*. Faulty agents have omission failures with more than *t* agents.

It is easy to see that *t* is the sum of the number of risk and faulty agents. We treat good agents and risk agents as nonfaulty agents uniformly. Send omission and receive omission are symmetrical. For example, the cases that *i* omits to send messages with *j* and that *j* omits to receive messages with *i* have the same view for *i* and *j*. Therefore, we may not be able to directly detect the states of some agents with omission failures. Thus, we call them risk agents and consider them as correct agents. For an agent that has omission failures with more than *t* agents, it must have omission failures with at least one good agent and then clearly we can know it is a faulty agent.

Due to the symmetry of agent omission failures, we model the agent omission failures as the link state problem by a punishment mechanism. Specifically, in our protocol, if an agent *i* receives no messages from *j* in a round, then in the following rounds, *i* sends no messages to *j* and does not receive messages from *j* [23]. Thus, both send omission or receive omission will cause the link interruption. So in a round, we divide each link link_*ij*_ into three types: correct link, where neither *i* nor *j* experiences omission failures in this round, faulty link, where at least one of *i* and *j* has omission failures with the other one in the round, and unknown − state link, where the state of link_*ij*_ in this round is unknown to another agent *k*. It is easy to see that we can determine the type of an agent by the number of correct direct links of it, which is the fault detection method in our protocol. Similarly, faulty links never recover under this punishment mechanism whether or not omission failures recover.

### 3.2. Consensus

In the consensus problem, we assume that every agent *i* has an initial preference *v*_*i*_ in a fixed value set *V* (we follow the concept of initial preference in [12]). We are interested in uniform consensus in this paper. A protocol solving uniform consensus must satisfy the following properties [3]:

- *Termination*. Every correct agent eventually decides.
- *Validity*. If an agent decides *v*, then *v* was the initial value of some agent.
- *Uniform Agreement*. No two agents (whether correct or not) decide on different values.

To solve uniform consensus in presence of agent omission failures, we assume that *n* > 2*t*+1 and *n* ≥ 3.

In uniform consensus, an agent's final decision must be one of the following formalized types:

- ⊥: it means that there is no consensus. ⊥ is a punishment for inconsistency.
- ||: it means no decision. Deciding || is not ambiguous with validity, as || cannot be proposed [23]. It does not affect the final consensus outcome.
- *v* ∈ *V*: it satisfies the property of validity, which must be the initial preference of some agent.

### 3.3. Rational Agent

We consider that distributed processes act as rational agents according to the definition in game theory. Each agent *i* has a utility function *u*_*i*_. We assume that agents have solution preference [18], and an agent's utility depends only on the consensus value achieved. Thus, for each agent *i*, there are three values of *u*_*i*_ based on the consensus value achieved: (i) *β*_0_ is *i*'s utility if *i*'s initial preference *v*_*i*_ is decided; (ii) *β*_1_ is *i*'s utility if there is a consensus value which is not equal to *i*'s initial preference; (iii) *β*_2_ is *i*'s utility if there is no consensus. It is easy to see that *β*_0_ > *β*_1_ > *β*_2_, and our results can easily be extended to deal with independent utility function for each agent.

The strategy of an agent *i* is a local protocol *σ*_*i*_ satisfying the system constrains. *i* takes actions according to the protocol *σ*_*i*_ in each round. That is, *σ*_*i*_ is a function from the set of messages received to actions. Each agent chooses the protocol in order to maximize its expected utility. Thus, there are *n* local protocols chosen by every agent, which is called $\text{strategy profile}\overset{⟶}{\sigma }$ in game theory. The equilibrium is a strategy profile, where each agent cannot increase its utility by deviating if the other agents fix their strategies. For each agent *i*, if the local protocol *σ*_*i*_ is our consensus algorithm when reaching an equilibrium, then we say that consensus is a Nash equilibrium and the consensus reaching a Nash equilibrium is called rational consensus. Formally, if a strategy profile (or consensus) $\overset{⟶}{\sigma }$ is a Nash equilibrium, then for all agents *i* and all strategies *σ*_*i*_′ for *i*, it must have $u_{i}\left(\sigma_{i}^{\prime },\;{\overset{⟶}{\sigma }}_{-i}\right)\leq u_{i}\left(\overset{⟶}{\sigma }\right)$.

### 3.4. Notation Description

The main notations used in following sections are summarized in Table 1.

## 4. Rational Uniform Consensus with General Omission Failures

### 4.1. A Rational Uniform Consensus in Synchronous Systems

In order to reach rational uniform consensus that can tolerate omission failures, our protocol adopts a simple idea from an early consensus protocol [23]: An agent does not send or receive any messages to those agents that did not send messages to it previously. Then, we convert the omission failure model which cannot be detected into the link state model which can be detected by agents in each round. However, the presence of rational agents makes protocol more complicated. It requires the protocol to prevent the manipulation of rational agents. Hence, the security of the algorithm needs to be improved from three aspects. The first is interacting with the latest network link states and message sources in each round. The update process of the latest link states within each agent depends on complete message chains, and we can obtain a unified decision round and decision set from message passing mechanism in omission failure environment. The second is using secret sharing for agents' initial preferences [24]. It encrypts the initial preferences so as to prevent an agent knowing the values of other agents in advance. The third is signing each message with a random number and marking faulty links by faulty random numbers [4]. This can improve the difficulty of a rational agent to do evil.

The protocol is described in Algorithm 1. In more detail, we proceed as follows.

Initially, each agent *i* generates a random number proposal_*i*_ which is used for consensus election later (line 2). Then *i* computes two random 1-degree polynomials *q*_*i*_ and *b*_*i*_ with *q*_*i*_(0) = *v*_*i*_ and *b*_*i*_(0) = proposal_*i*_, respectively (line 3). They satisfy (2, *n*) threshold which means that an agent *j* ≠ *i* can restore *v*_*i*_ or proposal_*i*_ if it knows more than two pieces of *q*_*i*_ or *b*_*i*_. Then *i* initialize set lost_*i*_, *NS*_*i*_^0^, *HS*_*i*_, decision_*i*_ and consensus_*i*_ (line 4); we discuss these in more detail below. Then *i* generates the faulty random number *X*-random_*i*_^1^[*k*][*l*_*ij*_] for each agent *k* ≠ *i* and each direct link *l*_*ij*_ that is the abbreviation of link_*ij*_ for the link between *i* and *j* (lines 5–7; *l*_*ij*_^*r*^ represents the link_*ij*_ in round *r*). And the message random number random_*i*_^1^ for round 1 is randomly chosen from {0,…, *n* − 1} (line 8). For each link, *i* generates *n* − 1 faulty random numbers and then sends them to other agents, respectively, in round 1. So, we can get that *X*-random_*i*_^1^ contains (*n* − 1)^2^ faulty random numbers in total. Then *i* puts *X*-random_*i*_^1^ and random_*i*_^1^ into X-RANDOM and RANDOM, respectively (lines 9 and 10), where X-RANDOM is a function storing all faulty random numbers known to *i* and RANDOM stores all message random numbers. Agents can invoke these two functions to verify random numbers. Specifically, input the id, link, and round to invoke X-RANDOM and input id and round to invoke X-RANDOM.

There are *t* + 4 rounds in total and each round has three phases. In phase 1 of round *r*, 1 ≤ *r* ≤ *t* + 4, *i* only sends messages to each agent *j* who does not belong to lost_*i*_ that is a set of agents that have omission failures with *i* detected by *i* (line 15). If 1 ≤ *r* ≤ *t* + 3, *i* sends random_*i*_^*r*^ and *NS*_*i*_^*r*−1^ to *j*. And if1 ≤ *r* ≤ *t* + 2, *i* also sends *X*-random_*i*_^*r*^[*j*] which contains *n* − 1 random numbers (lines 16 and 17). If *r* = 1, *i* also sends the piece of *q*_*i*_, *q*_*i*_(*j*) and the piece of *b*_*i*_, *b*_*i*_(*j*) to *j* (line 16). If *r* = *t* + 3, *i* also sends all the secret shares *q*_*l*_(*i*) and *b*_*l*_(*i*)(*l* ≠ *j*) that it has received from other agents (line 18). It is easy to see that the piece *q*_*l*_(*i*) and *b*_*l*_(*i*) must be in pairs. That is, if *i* restores *v*_*j*_, then it can also restore proposal_*j*_. Finally, if *r* = *t* + 4, *i* only sends consensus_*i*_ to *j* (line 19). For each agent *i*, consensus_*i*_ is the set of all consensus values calculated and received by *i*. Hence, if the algorithm is executed validly, |consensus_*i*_| must be equal to 1.

In phase 2 of round *r*, 1 ≤ *r* ≤ *t* + 4, *i* only receives messages from agents that are not in set lost_*i*_ (line 22). And if there are no messages received from an agent *j*, *j* ∉ lost_*i*_, *i* adds *j* to lost_*i*_ (line 29). Otherwise, *i* stores the received information (lines 24–28). {*NS*^*r*−1^} and {random^*r*^} are the sets of all new link states *NS*_*j*_^*r*−1^ and message random numbers random_*j*_^*r*^, respectively, received by *i* from each agents *j* ∉ lost_*i*_ in round *r* (line 26). Correspondingly, the elements in {*NS*^*r*−1^} and {random^*r*^} are one-to-one correspondence. Specially, if |lost_*i*_| > *t*, *i* knows that it becomes a faulty agent and then *i* must decide || directly and no longer run in later rounds (line 31). And we say that || means agent *i* does not decide in the end, which has no influence on the solution.

In phase 3 of round *r* ≤ *t* + 3, *i* firstly uses *NS*_*i*_^*r*−1^ to update *NS*_*i*_^*r*^ which is useful for the update and verification of link states (line 35). Then *i* invokes the function VERIFYANDUPDATE to verify and update *NS*_*i*_^*r*^ and *HS*_*i*_ by {*NS*^*r*−1^} and {random^*r*^} (line 36; see Algorithm 2 for details). *NS*_*i*_^*r*^ is the latest state known to *i* of all links in the system in round *r*. *HS*_*i*_ is the historical link state including *t* + 3 rounds in total. If *r* ≤ *t* + 2, *i* generates the message random number random_*i*_^*r*+1^ and the faulty random numbers *X*-random_*i*_^*r*+1^ for round *r* + 1, which will be sent to other agents in round *r* + 1, and then puts these random numbers into X-RANDOM and RANDOM, respectively (lines 37–43). Then if *r* = *t* + 3, *i* last updates *HS*_*i*_ by *NS*_*i*_^*t*+3^ (line 45). Specifically, if a link *l*_*kp*_ is faulty in round *m* in *NS*_*i*_^*t*+3^, then change the state of *l*_*kp*_ into fault from round *m* to round *t* + 3 in *HS*_*i*_. This is the last time modifying *HS*_*i*_. And following that, *i* utilizes *HS*_*i*_ to find the decision round *m*^*∗*^ from round 1 to round *t* + 2, which is the first reliable round in *HS*_*i*_ (lines 46–48). We follow the concept of clean round in [12]. The number of faulty agents does not increase in clean round and the previous round of clean round is reliable round. Specially, we say that reliable round cannot be round 0, so that the first reliable round is the previous round of the second clean round if the first clean round is round 1. In *HS*_*i*_^*r*^, if less than *n* − *t* − 1 links to agent *j* are correct, then *j* is a faulty agent. Otherwise, *j* is a nonfaulty agent. We define that it must remove the explicitly faulty agents when computing the state of *j* in round *r* by *HS*_*i*_. Then *i* computes the decision set *D* that is the set of nonfaulty agents in *HS*_*i*_^*m*^*∗*^^ (line 49). And then *i* uses all the secret shares it has received in round *t* + 3 to try to restore the initial preference and proposal of each agent *j* ∈ *D* (lines 50–53). If *i* can reconstruct the values of all agents ∈*D*, then *i* must know all proposals of these agents. Then *i* computes the consensus proposal (lines 54–63). Firstly, *i* sorts all the proposals in *D* and finds the set *C* of agents with the second max proposal value (line 55). Then if there is only one agent in *C*, *i* puts the initial preference of this agent into consensus_*i*_ (line 56). If there are more than one agents in *C*, that is, more than one agents have the same second max proposal value *pr* in *D* (we say that the probability is extremely low), then *i* uses the *pr* to mod the agent number of *C* and gets *S* (lines 60–62). In this case, *i* finally puts *v*_*j*_ into its consensus_*i*_ where *j* is the (*S* + 1) st highest id in *C* (line 63). Finally, if there is no agent in *C*, then the proposals must be the same for all agents in *D* and the second max proposal value does not exist. Thus, *i* uses the same proposal to mod the agent number of decision set *D* and gets *S* (line 58). Similarly, *i* elects the initial preference of the agent with the (*S* + 1) st highest id in *D* (line 59). But if *i* cannot restore all the values of agents ∈*D*, it does nothing and keeps consensus_*i*_ as the empty set. Finally, if *r* = *t* + 4, if consensus_*i*_ contains only one value, then *i* makes a decision (lines 65–67). Otherwise, an inconsistency is detected and *i* decides ⊥ (line 69).

The detailed implementation of the verification and update protocol in phase 3 is given in Algorithm 2.

Basically, for each link *l*_*kp*_, *NS*_*i*_^*r*^[*l*_*kp*_] is a tuple containing two tuples, *t*_*A*_ and *t*_*B*_. The first tuple *t*_*A*_ represents the state of *l*_*kp*_, which contains three types: *Msg*-*R*, *Msg*-*X* and *Msg*-*O*, representing correct link, faulty link, and unknown-state link, respectively. The format of type *Msg*-*R* is (*m*, *k*, random_*p*_^*m*^), where *m* is the round of the link state, *k* is the agent reporting the link state, and random_*p*_^*m*^ is the message random number sent by *p* in round *m*. It is easy to see that if *k* reports the state of its direct link *l*_*kp*_ is correct (*Msg*-*R*) in round *m*, then it must knowrandom_*p*_^*m*^. The format of type *Msg*-*X* is (*X*, *m*, *k*, *X*-random_*k*_^*m*^[*l*_*kp*_]), where *m* and *k* are the same as those in *Msg*-*R*, *X* is an identifier, and *X*-random_*k*_^*m*^[*l*_*kp*_] is the sorted set of faulty random numbers on *l*_*kp*_ which is generated by *k* in round *m*-1. Specifically, the set is sorted by the ids of agents from small to large. The format of type *Msg*-*O* is ∅ because the state of *l*_*kp*_ is unknown for *i*. The second tuple *t*_*B*_ describes the source of *t*_*A*_ and has the form (*j*, *m*), where *j* is the agent sending the link state *t*_*A*_ to *i*, and *m* is the round when *j* sends it to *i*. Specially, for direct link, when *i* first updates the state in round *r*, *t*_*B*_ is *ϕ* meaning that the message source is *i* itself. *HS*_*i*_^*r*^[*l*_*kp*_] denotes the state of link *l*_*kp*_^*r*^ known to *i* and *r* could range from 1 to *t* + 3. It contains at most two different tuples because the state of *l*_*kp*_ in round *r* can only be detected and reported by *k* and *p*. The form of each tuple is similar to *t*_*A*_ but the round in *t*_*A*_ must be *r*. And the agents in the two tuples must be different and be *k* and *p*, respectively. Specially, if the types of two tuples are *Msg*-*R* and *Msg*-*X*, then we think the state of *l*_*kp*_^*r*^ is faulty. And if *Msg*-*O* and *Msg*-*O*, then *l*_*kp*_^*r*^ is a unknown-state link which is regarded as a correct link when computing decision round and decision set in round *t* + 3.

The pseudocode in Algorithm 2 is explained in detail as follows.

*i* initially generates*T* from {*NS*^*r*−1^}, which is easy to see that *i* must receive the messages sent by agent *j* ∈ *T* in round *r* (line 2). And *i* also computes set *S* that is equal to lost_*i*_ (line 3).

Firstly, in phase 1, *i* updates the states of direct links in round *r*. For each agent *j* ∈ *T*, *i* has received the messages from it in round *r* so that *i* updates the *t*_*A*_ of *NS*_*i*_^*r*^[*l*_*ij*_] to Type *Msg*-*R* (line 7). And *i* must be able to obtain the message random number random_*j*_^*r*^ from {random^*r*^}. Then *i* invokes APPENDHS to append the state (*r*, *i*, random_*j*_^*r*^) into *HS*_*i*_^*r*^[*l*_*ij*_] (line 8). We stipulate APPENDHS must guarantee that the inputting state satisfies the properties of *HS* which we have discussed above. For example, each link *l*_*kp*_ has at most two different tuples in each round, and they come from different agents, *k* and *p*. If a state violated the properties of *HS*, APPENDHS would decide ⊥ and terminate the protocol early. Then for each agent *j* ∈ *S*, it has omission failures detected by *i* because *i* does not receive a message from it. If the type of link *l*_*ij*_ is already faulty in *NS*_*i*_^*r*^ inherited *NS*_*i*_^*r*−1^, *i* does nothing because for a link, *NS* only records the earliest round when the link has failures (lines 10–11). Otherwise, *i* updates the *t*_*A*_ to Type *Msg*-*X* and appends the new state into *HS*_*i*_ (lines 13–14).

Then in phase 2, *i* utilizes message chain mechanism to verify the correctness of messages {*NS*^*r*−1^} received in receive phase (lines 17–18).

*Message Chain Mechanism*. For each agent *j*, its message *NS*_*j*_^*m*^ has the following properties.

Suppose *S*_*j*_^*m*^ is the set of agents that disconnected from *j* in or before round *m* and *T*_*j*_^*m*^ is the set of agents that are still connected to *j* in round *m*. Suppose *X*(*r*) represents the *Msg* − *X* tuple where the round number is equal to *r*.

**Claim 1 .**

For link *l*_*kp*_ in *NS*_*j*_^*m*^, where *k*=*j* and *p* ∈ *S*_*j*_^*m*^ ∪ *T*_*j*_^*m*^, its state in round *m* must be known and the number of correct links in {*l*_*jp*_} is greater than or equal to *n* − *t* − 1.

**Claim 2 .**

For link *l*_*kp*_ in *NS*_*j*_^*m*^, where *k*=*j* and *p* ∈ *S*_*j*_^*m*^ ∪ *T*_*j*_^*m*^, its state in round *m*+1 and later must be unknown.

**Claim 3 .**

For link *l*_*kp*_ in *NS*_*j*_^*m*^, where *k* ∈ *S*_*j*_^*m*^ and *p* ∈ *S*_*j*_^*m*^, its state in round *m* − 1 and later must be unknown.

**Claim 4 .**

For link *l*_*kp*_ in *NS*_*j*_^*m*^, where *k* ∈ *S*_*j*_^*m*^ and *p* ∈ *S*_*j*_^*m*^, if the state of *NS*_*j*_^*m*^[*l*_*jk*_] is *X*(*m*_1_) and the state of *NS*_*j*_^*m*^[*l*_*jp*_] is *X*(*m*_2_), suppose *m* ≥ *m*_1_ ≥ *m*_2_, then the state of *l*_*kp*_^*m*_1_−2^ must be known.

**Claim 5 .**

For link *l*_*kp*_ in *NS*_*j*_^*m*^, where *k* ∈ *T*_*j*_^*m*^ and *p* ∈ *S*_*j*_^*m*^ ∪ *T*_*j*_^*m*^, its state in round *m* − 1 must be known and that in round *m* and later must be unknown.

**Claim 6 .**

For link *l*_*kp*_ in *NS*_*j*_^*m*^, where *k* ∈ *T*_*j*_^*m*^ and *p* ∈ *S*_*j*_^*m*^, if the state of *l*_*kp*_^*m*−1^ is *Msg* − *R*, then the state of link *l*_*pt*_ in round *m* − 2, where *t* ∈ *S*_*j*_^*m*^, must be known and its state in round *m* − 1 and later must be unknown.

**Claim 7 .**

For link *l*_*kp*_ in *NS*_*j*_^*m*^, where *k* ∈ *T*_*j*_^*m*^ and *p* ∈ *S*_*j*_^*m*^, if the state of *l*_*kp*_^*m*−1^ is equal to*X*(*m*′), where *m*′ ≤ *m* − 1, then the state of *l*_*pt*_^*m*′−2^, *t* ∈ *S*_*j*_^*m*^, must be known.

Explicitly, we say that the function VERIFYMSGCHAIN is to verify whether a message *NS*_*j*_^*r*−1^ ∈ {*NS*^*r*−1^} violates the above claims. If not, then continue to the phase 3. Otherwise, it decides ⊥ and terminates the protocol early.

Finally, in phase 3, *i* updates *NS*_*i*_^*r*^ and *HS*_*i*_ by the states in {*NS*^*r*−1^}. For a link, *i* compares its state in *NS*_*i*_^*r*^ with the state in *NS*_*j*_^*r*−1^ ∈ {*NS*^*r*−1^}, so as to implement update according to different cases.

**Claim 8 .**

For each link *l*_*kp*_(*k*, *p* ∈ *N*), the agent of *t*_*A*_ must be *k* or *p* in all *NS*[*l*_*kp*_] and *HS*[*l*_*kp*_].

 
*Case 1*. For the direct link *l*_*ij*_ of *i*, if the *t*_*A*_ of *NS*_*i*_^*r*^[*l*_*ij*_] is (*r*, *i*, random) and the *t*_*A*_ of *NS*_*j*_^*r*−1^[*l*_*ij*_] is (*r*′, *k*, random′), then *i* only needs to append the new state into *HS*_*i*_ (lines 39–40).

**Claim 9 .**

In Case 1, there must be *r*′ < *r*.

 
*Case 2*. For the direct link *l*_*ij*_ of *i*, if the *t*_*A*_ of *NS*_*i*_^*r*^[*l*_*ij*_] is (*r*, *i*, random) and the *t*_*A*_ of *NS*_*j*_^*r*−1^[*l*_*ij*_] is (*X*, *r*′, *k*, *X*-random_*k*_^*r*′^[*l*_*ij*_]),then *i* detects an inconsistency and decides ⊥ (lines 41–42).
 
*Case 3*. For the direct link *l*_*ij*_ of *i*, if the *t*_*A*_ of *NS*_*i*_^*r*^[*l*_*ij*_] is (*X*, *r*′, *k*, *X*-random_*k*_^*r*′^[*l*_*ij*_])and the *t*_*A*_ of *NS*_*j*_^*r*−1^[*l*_*ij*_] is (*r*^″^, *p*, random), then *i* only needs to append the new state into *HS*_*i*_ (lines 43–44).

**Claim 10 .**

In Case 3, there must be *r*^″^ ≤ *r*′.

 
*Case 4*. For the direct link *l*_*ij*_ of *i*, if the *t*_*A*_ of *NS*_*i*_^*r*^[*l*_*ij*_] is (*X*, *r*′, *i*, *X*-random_*i*_^*r*′^[*l*_*ij*_])and the *t*_*A*_ of *NS*_*j*_^*r*-1^[*l*_*ij*_] is (*X*, *r*^″^, *k*, *X*-random_*k*_^*r*^″^^[*l*_*ij*_]),then *i* only needs to append the new state into *HS*_*i*_ when *r*^″^ = *r*′ or *r*^″^ = *r*′ + 1, and *i* must update *NS*_*i*_^*r*^ and *HS*_*i*_ when *r*^″^ = *r*′-1 (lines 45–51). When updating *NS*_*i*_^*r*^, the *t*_*B*_ of *NS*_*i*_^*r*^[*l*_*ij*_] must be (*j*, *r*) because the new state is obtained from *NS*_*j*_^*r*−1^ and updated in round *r*.

**Claim 11 .**

In Case 4, if *k* = *i*, the *t*_*A*_ of *NS*_*j*_^*r*−1^[*l*_*ij*_] must be the same as the *t*_*A*_ of *NS*_*i*_^*r*^[*l*_*ij*_], and if *k* = *j*, it must have 0 ≤ |*r*′ − *r*^″^| ≤ 1.

 
*Case 5*. For the direct link *l*_*ij*_ of *i*, if the *t*_*A*_ of *NS*_*i*_^*r*^[*l*_*ij*_] is (*X*, *r*′, *j*, *X*-random_*j*_^*r*′^[*l*_*ij*_]) and the *t*_*A*_ of *NS*_*j*_^*r*−1^[*l*_*ij*_]is (*X*, *r*^″^, *k*, *X*-random_*k*_^*r*^″^^[*l*_*ij*_]),then *i* does nothing.

**Claim 12 .**

In Case 5, if *k* = *j*, the *t*_*A*_ of *NS*_*j*_^*r*−1^[*l*_*ij*_] must be the same as the *t*_*A*_ of *NS*_*i*_^*r*^[*l*_*ij*_], and if *k* = *i*, it must have *r*^″^ = *r*′ + 1.

 
*Case 6*. For the indirect link *l*_*kp*_ of *i*, if the *t*_*A*_ of *NS*_*i*_^*r*^[*l*_*kp*_] is (*r*^″^, *y*, random′) and the *t*_*A*_ of *NS*_*j*_^*r*−1^[*l*_*kp*_] is (*r*^″^, *z*, random′),then *i* only needs to append the new state into *HS*_*i*_ when *r*^″^ ≤ *r*′, and *i* must update *NS*_*i*_^*r*^ and *HS*_*i*_ when *r*^″^ > *r*′ (lines 53–58).
 
*Case 7*. For the indirect link *l*_*kp*_ of *i*, if the *t*_*A*_ of *NS*_*i*_^*r*^[*l*_*kp*_] is (*r*′, *y*, random) and the *t*_*A*_ of *NS*_*j*_^*r*−1^[*l*_*kp*_] is (*X*, *r*^″^, *z*, *X*-random_*z*_^*r*^″^^[*l*_*kp*_]),then *i* needs to update *NS*_*i*_^*r*^ and append the new state into *HS*_*i*_ (lines 59–60).

**Claim 13 .**

In Case 7, if *z* = *y*, it must have *r*′ < *r*^″^, and if *z* ≠ *y*, it must have *r*′ ≤ *r*^″^.

 
*Case 8*. For the indirect link *l*_*kp*_ of *i*, if the *t*_*A*_ of *NS*_*i*_^*r*^[*l*_*kp*_] is (*X*, *r*′, *y*, *X*-random_*y*_^*r*′^[*l*_*kp*_])and the *t*_*A*_ of *NS*_*j*_^*r*−1^[*l*_*kp*_] is (*r*^″^, *z*, random), then *i* only needs to append the new state into *HS*_*i*_ (lines 62–63).

**Claim 14 .**

In Case 8, if *z* = *y*, it must have *r*′ > *r*^″^, and if *z* ≠ *y*, it must have *r*′ ≥ *r*^″^.

 
*Case 9*. For the indirect link *l*_*kp*_ of *i*, if the *t*_*A*_ of *NS*_*i*_^*r*^[*l*_*kp*_] is (*X*, *r*′, *y*, *X*-random_*y*_^*r*′^[*l*_*kp*_]))and the *t*_*A*_ of *NS*_*j*_^*r*−1^[*l*_*kp*_] is (*X*, *r*^″^, *z*, *X*-random_*z*_^*r*^″^^[*l*_*kp*_]),then *i* does nothing when *z* = *y*, and *i* appends the new state into *HS*_*i*_ when *z* ≠ *y*. Specially, *i* also updates *NS*_*i*_^*r*^ using the new state received if *r*′ > *r*^″^ (lines 64–70).

**Claim 15 .**

In Case 9, if *z* = *y*, the *t*_*A*_ of *NS*_*j*_^*r*−1^[*l*_*kp*_] must be the same as the *t*_*A*_ of *NS*_*i*_^*r*^[*l*_*kp*_], and if *z* ≠ *y*, it must have 0 ≤ |*r*′ − *r*^″^| ≤ 1.

 
*Case 10*. If the *t*_*A*_ of *NS*_*j*_^*r*−1^[*l*_*kp*_] is ∅, then *i* does nothing (lines 28–29).
 
*Case 11*. For the indirect link *l*_*kp*_ of *i*, if the *t*_*A*_ of *NS*_*i*_^*r*^[*l*_*kp*_] is ∅ and the *t*_*A*_ of *NS*_*j*_^*r*−1^[*l*_*kp*_] is (*r*′, *z*, random) or (*X*, *r*′, *z*, *X*-random_*z*_^*r*′^[*l*_*kp*_])),then *i* needs to update *NS*_*i*_^*r*^ and append the new state into *HS*_*i*_. (lines 30–32).

**Claim 16 .**

If in round *r*, agent *i* receives a message in which *t*_*B*_ is (*j*, *m*) and *j* ∈ *T*_*i*_^*m*^ or *j*=*i*, then *t*_*A*_ of the message must already be in *HS*_*i*_ when in round *r*.

**Claim 17 .**

If in round *r*, agent *i* receives a message in which *t*_*B*_ is (*j*, *r* − 1) from *k*, then Type(*NS*_*k*_^*r*−1^[*l*_*kj*_^*r*−1^]) must be *Msg*-*R*.

In phase 3, for a link *l*_*kp*_, *i* needs to detect whether there is an inconsistency firstly (line 26). An inconsistency detected in phase 3 may be because

1. (message format verification). The format of *NS*_*j*_^*r*−1^[*l*_*kp*_] is incorrect;
2. (message source verification). *NS*_*j*_^*r*−1^[*l*_*kp*_] violates Claim 16 or Claim 17;
3. (random number verification). If the type of *NS*_*j*_^*r*−1^[*l*_*kp*_] is *Msg*-*R*, the message random number in *NS*_*j*_^*r*−1^[*l*_*kp*_] is different from that in Random, or if *Msg*-*X*, the faulty random numbers in X-RANDOM are different from the random numbers at the corresponding indexes of the sorted set in *NS*_*j*_^*r*−1^[*l*_*kp*_];
4. (round number verification). *NS*_*j*_^*r*−1^[*l*_*kp*_] violates one of the claims from Claim 8 to Claim 15.

If *i* detects an inconsistency, then it decides ⊥ (line 27). If not, *i* updates the states as previously discussed.

### 4.2. Proof of the Protocol

The proof assumes *n* > 2*t*+1. Some variables are defined as follows.

**Definition 1 .**

State_*i*_[*l*_*ij*_^*r*^] denotes the detection result of agent *i* on the state of direct link *l*_*ij*_ in round *r*. The type of State_*i*_[*l*_*ij*_^*r*^] must be *Msg*-*R* or *Msg*-*X*.

**Definition 2 .**

*C*_*i*_^*j*^(*m*_1_, *m*_2_)denotes the agent chain (or we can call it message propagation path) from agent *i* to *j*. *i* detects a direct link state in round *m*_1_ and sends it to agent *k* ≠ *i*, *j* in round *m*_1_ + 1. Then *k* also sends the state to another agent in round *m*_1_ + 2. Finally, *j* receives the state in round *m*_2_.

**Definition 3 .**

*Nf* ^*r*^ denotes the set of nonfaulty agents in round *r*. *F*^*r*^ denotes the set of faulty agents in round *r*. *F*^Δ*r*^ denotes the set of faulty agents newly detected in round *r*. *x*_*r*_ denotes the number of risk agents in round *r*.

We first prove the upper bound of message passing time and give the round complexity of the algorithm.

**Theorem 1 .**

(message passing mechanism). If *i*, *j* ∈ *Nf*^*r*+*t*+1^, all link states in round *r* can be reached a consensus between *i* and *j* at the latest in round *r*+*t*+1.

**Proof**

Consider the state of link_*kp*_ in round *r*, where *k*, *p* ∈ *N*. Specially, we can consider the messages sent by *k* and *p* to be independent of each other and this does not affect the final consensus outcome. For example, Type(State_*k*_[*l*_*kp*_^*r*^]) = *Msg*-*X* and it is received by all nonfaulty agents in round *m*_1_(<*r* + *t* + 1), and Type(State_*p*_[*l*_*kp*_^*r*^]) = *Msg*-*R* and it is received by all nonfaulty agents in round *m*_2_(*m*_1_ < *m*_2_ < *r* + *t* + 1). Even if the detection result of *p* may no longer be forwarded after round *m*_1_, we still have the correct consensus state in round *r* + *t* + 1 when we consider two detection results independently. We have following cases:

(i) *Case 1*. *k* and *p* are good agents in round *r*. In round *r* + 1, *k* and *p* send their detection results to all good agents. So if *t* = 0, all agents reach a consensus on the state of *l*_*kp*_ in round *r* + 1. If *t* > 0, all nonfaulty agents reach a consensus in round *r* + 2. Therefore, all link states of round *r* among good agents can reach a consensus in round *r* + *t* + 1.
(ii) *Case 2*. *k* is a risk agent or faulty agent and *p* is not equal to *k*. Generally, since a receive omission can be converted to a send omission, then each risk agent and faulty agent has at most the following 3 choices when sending messages in each round:
  1. It has sending omissions with all other agents.
  2. It does not have sending omissions with at least a good agent.
  3. It has sending omissions with all good agents and no sending omissions with some risk agents or faulty agents.
Hence, *k* has three choices in round *r* + 1.
(1) *Case 2.1*. *k* chooses 1. Then all agents do not know State_*k*_[*l*_*kp*_^*r*^]. All nonfaulty agents agree on the “unknown-state.”
(2) *Case 2.2*. *k* chooses 2. Then there must be some good agents knowing State_*k*_[*l*_*kp*_^*r*^] in round *r* + 1. And all good agents and *k* know the state in round *r* + 2. If *t* = 1, *k* is the only risk agent, then there is a consensus on the state in round *r* + 2. But if *t* > 1, all nonfaulty agents receive State_*k*_[*l*_*kp*_^*r*^] in round *r* + 3 because all good agents must send it to all nonfaulty agents in this round. Thus, the lemma holds.
(3) *Case 2.3*. *k* chooses 3. So no good agents know State_*k*_[*l*_*kp*_^*r*^] in round *r* + 1 and *k* is detected faulty in round *r* + 1. Suppose that there is only a risk agent receiving the state. Since agents are independent of each other, it is easy to scale the number of the agents from one to many. We can also divide this case into two cases.
  - *Case 2.3.1*. *k* only sends messages to *p* in round *r* + 1. It means that Type(State_*k*_[*l*_*kp*_^*r*^]) = *Msg*-*R*. If Type(State_*p*_[*l*_*kp*_^*r*^]) = *Msg*-*X*, *p* does not receive messages from *k* in round *r* + 1. Then the result is the same as that in case 2.1. But if Type(State_*p*_[*l*_*kp*_^*r*^]) = *Msg*-*R*, *k* has no influence on the final result and the consensus result of *l*_*kp*_^*r*^ depends on the choice of *p*. If *p* also chooses Case 2.3.1, then two states of *l*_*kp*_^*r*^ only exist in *k* and *p*. The final result is also the same as that in case 2.1.
  - *Case 2.3.2*. *k* has no sending omissions with some risk agents or faulty agents other than *p*. Then in round *r* + 2, the risk agents and faulty agents that have received messages from *k* also have 3 choices. Take one of the risk agents *l* as an example. If *l* chooses 1 or 2 in round *r* + 2, the results are the same as those in case 2.1 and case 2.2 where the lemma holds. And if *l* chooses 3, no good agents know State_*k*_[*l*_*kp*_^*r*^] in round *r* + 2. Suppose that when risk and faulty agents choose 3, they must send State_*k*_[*l*_*kp*_^*r*^] to risk agents or faulty agents other than the source agent of the state because if they only send the state back to the source agent, the final results depend only on the source agent, not on themselves. Then until round *r* + *t*, if from round *r* + 1 to round *r* + *t* − 1, all risk agents and faulty agents that have received State_*k*_[*l*_*kp*_^*r*^] choose 3, then the risk (or faulty) agent *z* in round *r* + *t* must be the last risk (or faulty) agent in system. At this time, *z* has only 2 choices: 1 and 2. And it is easy to get that State_*k*_[*l*_*kp*_^*r*^] must be consensus in round *r* + *t* + 1. But if from round *r* + 1 to round *r* + *t* − 1, some risk agents or faulty agents that have received State_*k*_[*l*_*kp*_^*r*^] choose 1 or 2, then the results are the same as those in case 2.1 and case 2.2.

In summary, the lemma holds.

**Corollary 1 .**

If *i*, *j* ∈ *Nf*^*r*+*x*_*r*_+1^, all link states in round *r* can reach a consensus between *i* and *j* in round *r*+*x*_*r*_+1.

**Proof**

There are *t* − *x*_*r*_ faulty agents in round *r*. Since the faulty agents before round *r* do not send any messages in round *r* + 2, it is equivalent to case 2.1 that *k* sends messages to these faulty agents in round *r* + 1. Then the total number of risk and faulty agents in case 2.3 can be reduced to *x*_*r*_. Therefore, in case 2.3.2, if keeping choosing 3, there are no risk or faulty agents anymore up to round *r* + *x*_*r*_ and all link states in round *r* can be reached a consensus between *i* and *j* in round *r* + *x*_*r*_ + 1.

**Lemma 1 .**

(*round complexity*). The link states *HS* of the second clean round and all previous rounds can reach a consensus among all nonfaulty agents at the latest in round *t*+3.

**Proof**

By Theorem 1, the smaller the round *r*, the smaller the supremum of the round in which the link states in round *r* can reach a consensus. Hence, we directly consider the second clean round. Suppose the second clean round is *y*. Then there are already at least *y* − 2 faulty agents in round *y*. That is, *x*_*y*_ ≤ *t* − *y*+2. By Corollary 1, the link states in round *y* can reach consensus in round *y*+*x*_*y*_+1. Since *y*+*x*_*y*_+1 ≤ *t*+3, the lemma holds.

Then it is proved that the algorithm satisfies all the properties of uniform consensus with general omission failures.

**Lemma 2 .**

If *i* is a nonfaulty agent, then Type(*HS*_*i*_^*r*∼*t*+3^[*l*_*kp*_])=*Msg*-*X* when Type(*HS*_*i*_^*r*^[*l*_*kp*_])=*Msg*-*X*(*k*, *p* ∈ *N*).

**Proof**

Link *l*_*kp*_ cannot recover after a fault occurs. So if *l*_*kp*_ is a faulty link in round *r*, then its state must also be *Msg*-*X* in subsequent rounds. Moreover, *HS* also expands all *Msg*-*X* states backwards in LastUpdate.

**Lemma 3 .**

If *i* is a nonfaulty agent, then Type(*HS*_*i*_^1∼*r*−1^[*l*_*kp*_])=*Msg*-*R* when Type(*HS*_*i*_^*r*^[*l*_*kp*_])=*Msg*-*R*(*k*, *p* ∈ *N*).

**Proof**

Since Type(*HS*_*i*_^*r*^[*l*_*kp*_]) ≠ *Msg*-*O*, there must be an agent in *k* or *p* (supposing *p*) that has reported State_*p*_[*l*_*kp*_^*r*^] in round *r* + 1, and finally the state has been transmitted to *i*. We suppose that *i* receives the state in round *r*′. Then we have *C*_*p*_^*i*^(*r*, *r*′). Since link omission is irreversible, *C*_*p*_^*i*^(*r*, *r*′)must be nonfaulty from round 1 to round *r* − 1. Hence, State_*p*_[*l*_*kp*_^1∼*r*−1^] must eventually be received by *i*. That means Type(*HS*_*i*_^1∼*r*−1^[*l*_*kp*_]) ≠ *Msg*-*O*. Combining Lemma 2, it is easy to get Type(*HS*_*i*_^1∼*r*−1^[*l*_*kp*_]) ≠ *Msg*-*X*. Thus, the lemma holds.

**Lemma 4 .**

If *i* is a nonfaulty agent, then Type(*HS*_*i*_^*r*∼*t*+3^[*l*_*kp*_])=*Msg*-*O* when Type(*HS*_*i*_^*r*^[*l*_*kp*_])=*Msg*-*O*(*k*, *p* ∈ *N*).

**Proof**

For a contradiction, let Type(*HS*_*i*_^*m*_1_^[*l*_*kp*_]) ≠ *Msg*-*O*(*m*_1_ > *r*) when Type(*HS*_*i*_^*r*^[*l*_*kp*_]) = *Msg*-*O*. Suppose that *i* receives the state of *link*_*kp*_^*m*_1_^ in round *m*_2_(*m*_2_ > *m*_1_). Let us suppose *i* receives it from *k*. Then we must have *C*_*k*_^*i*^(*m*_1_, *m*_2_). Since link omission is irreversible, the message propagation path is also correct for round *r*, so that *i* must receive State_*k*_[*l*_*kp*_^*r*^] and then Type(*HS*_*i*_^*r*^[*l*_*kp*_]) ≠ *Msg*-*O*. Therefore, we have a contradiction here and the lemma holds.

**Lemma 5 .**

A nonfaulty agent must have correct links with at least *n* − *t* − 1 agents other than itself in a round.

**Proof**

We can know that for a nonfaulty agent *i*, |lost_*i*_| must be less than or equal to *t*. So it is easy to get that *i* have correct links with at least *n* − *t* − 1 agents.

**Lemma 6 .**

A nonfaulty agent must have correct links with at least 2 good agents other than itself in a round.

**Proof**

We analyze the nonfaulty agent *i* from two aspects of good agent and risk agent.

- *Case 1*. *i* is a good agent. Then *i* must have correct links with all other *n* − *t* − 1 good agents. Since *n* > 2*t*+1 and *n* ≥ 3, there must be *n* − *t* − 1 ≥ 2.
- *Case 2*. *i* is a risk agent. Suppose that *i* is nonfaulty in round *r* and there are *f* faulty agents in this round. Because it must remove faulty agents when computing the state of *i*, combining Lemma 5, the risk agent *i* needs to have correct links with at least (*n* − *t* − 1) − (*t* − *f* − 1)=*n* − 2*t*+*f* good agents in round *r*. Since *n* − 2*t* > 1, *n* − 2*t*+*f* > *f*+1. Then *n* − 2*t*+*f* ≥ 2 always holds.

Therefore, the lemma holds.

**Remark 1 .**

For a faulty agent *f* in round *r*, since it has faulty links with more than *t* agents in round *r*, then it does not send messages to any agents after at most 2 rounds. Hence, we claim that in round *r* and later, the faulty agent *f* needs to be removed when computing the number of connections of other agents.

**Lemma 7 .**

Suppose that the direct link state information of *j* in round *r* can be agreed by all nonfaulty agents in round *m*. If *i* is a nonfaulty agent in round *m* and agent *j* is considered to be a uncertain agent in *HS*_*i*_^*r*^, then *j* must be a faulty agent in *HS*_*i*_^*r*+1^.

**Proof**

The proof argument is by contradiction. Assume that *j* is considered to be a nonfaulty agent or a uncertain agent in *HS*_*i*_^*r*+1^.

- *Case 1*. *j* is a nonfaulty agent in *HS*_*i*_^*r*+1^ when it is considered to be a uncertain agent in *HS*_*i*_^*r*^. *j* must send *NS*_*j*_^*r*^ to at least 2 good agents in round *r*+1 (Lemma 6). Then these good agents send the direct link states of *j* to all nonfaulty agents. Hence, *j* must be a certain agent in *HS*_*i*_^*r*^. A contradiction.
- *Case 2*. *j* is a uncertain agent in *HS*_*i*_^*r*+1^ when it is considered to be a uncertain agent in *HS*_*i*_^*r*^. It is easy to see that the link states between *j* and good agents cannot be unknown-state in round *r* and *r*+1. Since the number of good agents *n* − *t* must be greater than *t*, *j* cannot have faulty links with all good agents. Then it must send *NS*_*j*_^*r*^ to some good agents in round *r*+1. Equally, *j* must be a certain agent in *HS*_*i*_^*r*^. A contradiction.

Thus, we reach contradictions in all cases, which proves the lemma.

**Lemma 8 .**

If round *r* is a clean round, the state of *l*_*ij*_^*r*−1^ can reach a consensus by all nonfaulty agents in round *r*+2, where *i*, *j* ∈ *Nf*^*r*−1^ and *j* ≠ *i*.

**Proof**

We can pay attention to the state of *l*_*ij*_^*r*−1^. Consider the following cases:

- *Case 1*. *i* and *j* are good agents. By Lemma 6, a risk agent must have correct links with some good agents. Hence, *i* sends *State*_*i*_[*l*_*ij*_^*r*−1^] to all good agents and risk agents having correct links with *i* in round *r*. And *j* also does this. In round *r* all good agents have two detection results of *l*_*ij*_^*r*−1^. Then after updating, they send the uniform state to all risk agents that have faulty links with *i* and *j* in round *r*+1.
- *Case 2*. *i* and *j* are risk agents. *i* and *j* send their detection results of *l*_*ij*_^*r*−1^ to some good agents (denoted by *U*) and risk agents in round *r*. Then two results are sent to all good agents by the agents in *U* in round *r*+1. So every good agent knows the uniform state of *l*_*ij*_^*r*−1^ in round *r*+1. Therefore, all nonfaulty agents reach a consensus on the state in round *r*+2.
- *Case 3*. *i* is a good agent and *j* is a risk agent. Similarly, it is easy to get that all good agents have the uniform state of *l*_*ij*_^*r*−1^ in round *r*+1 by case 1 and case 2. So this is what we want. Thus, the lemma holds.

**Lemma 9 .**

If round *r* is a clean round, then in the *HS*_*i*_^*r*−1^(*i* ∈ *Nf*^*r*+2^), the state of link *l*_*kp*_ (*k* ∈ *Nf*^*r*−1^ and *p* ∈ *N*) cannot be *Msg* − *O*.

**Proof**

Assume, without influence, that the messages of *p* have no effect on the state of *l*_*kp*_. Since *k* is a nonfaulty agent, by Lemma 8, State_*k*_[*l*_*kp*_^*r*−1^] is received by all nonfaulty agents in round *r*+2. Hence, *i* must know the state of *l*_*kp*_^*r*−1^. The lemma holds.

**Corollary 2 .**

If round *r* is a reliable round and the total number of rounds is greater than *r*+3, there are not uncertain agents in round *r*.

**Proof**

For a contradiction, let *i* be a uncertain agent in round *r*, by Lemma 7, we have two cases. For both case 1 and case 2, by Lemma 8, there are contradictions to the assumption. Hence, *i* must be a faulty agent in *HS*^*r*+1^. The unknown-state link is regarded as correct link so that *i* is regarded as a nonfaulty agent in *HS*^*r*^. Then it is a contradiction to the assumption that *r* is a reliable round. Thus, the lemma holds.

**Lemma 10 .**

There is at least one clean round in *t*+1 rounds.

**Proof**

Suppose, for a contradiction, that there is no clean round in *t*+1 rounds. Then there must be new faulty agents added in each round. So there are at least *t*+1 faulty agents in *t*+1 rounds. This contradicts the assumption that there are at most *t* faulty agents.

**Corollary 3 .**

There are at least two clean rounds in *t*+2 rounds.

**Corollary 4 .**

In *t*+2 rounds, there must be one reliable round *r* in which at most one new faulty agent is detected.

**Proof**

Suppose that there are *a* clean rounds in *t*+2 rounds. We prove the lemma from two cases:

- *Case 1*. All clean rounds are greater than round 1. And for a contradiction, two new faulty agents are detected in each reliable round. Then there are 2*a* faulty agents and there are still *t* − 2*a* faulty agents remaining in *t*+2 − 2*a* rounds. It is easy to see that *t*+2 − 2*a* > *t* − 2*a*. Therefore, there must be clean rounds in the remaining *t*+2 − 2*a* rounds. This contradicts the assumption that there are *a* clean rounds in *t*+2 rounds.
- *Case 2*. Round 1 is a clean round. And for a contradiction, two new faulty agents are detected in each reliable round. Then there are 2(*a* − 1) faulty agents and there are still *t* − 2*a*+2 faulty agents remaining in *t*+2 − 2*a*+1 rounds. Since *t*+2 − 2*a*+1 > *t* − 2*a*+2, there must be clean rounds in the remaining *t*+2 − 2*a*+1 rounds, a contradiction.

Thus, we reach a contradiction in every case, which proves the lemma.

**Lemma 11 .**

In round *t*+3, if a faulty agent *i* ∈ *F*^Δ*t*+3^ can receive messages from at least one good agent, the link states of the second clean round and all previous rounds can also reach a consensus among *i* and all nonfaulty agents.

**Proof**

By Corollary 4, from the second clean round *y* to round *t*+3, there must be a reliable round (suppose the first is *r*) in which at most one new faulty agent is detected because the total number of risk and faulty agents is *t* − *y*+2 and the total number of rounds is *t* − *y*+4. Suppose *r*+1=*y*′ which is a clean round. Since *F*^Δ*t*+3^ ≠ ∅, *y* < *y*′<*t*+3. Since no new faulty agents are detected in round *y*′, risk agents can only choose 2 in round *y*′ (see details in Theorem 1). Hence, in round *y*′+1, all good agents reach a consensus on the link states *HS* of round *y* and before rounds. We divide *y*′ into two cases to prove as follows:

- *Case 1*. *y* < *y*′ < *t*+2. Then we have *y*′+2 ≤ *t*+3. In round *y*′+2, all good agents send the latest and uniform link states of round *y* and before rounds to all agents. Thus, *i* must reach a consensus.
- *Case 2*. *y*′=*t*+2. We assume that for the reliable rounds in which two or more faulty agents are detected, the faulty agents can be averaged to the next round and then the clean round can also be regarded as a normal round. Then it can be seen that the number of faulty agents keeps increasing in each round from round *y*+1 to round *t*+1. Thus, at least *t*+1 − *y* faulty agents have been added until round *y*′. Since there are *x*_*y*_(≤*t* − *y*+2) risk agents in round *y*, then at most one risk agent remains in round *y*′ and it must be *i*. Then it is easy to get see *i* must reach a consensus in round *t*+3.

Thus, the lemma holds.

**Theorem 2 .**

Consensus solves uniform consensus if at most *t* agents omit to send or receive messages, *n* > 2*t*+1, and suppose that all agents are honest.

**Proof**

Since *n* > 2*t*+1, it is easy to see that no inconsistency is detected.

*Termination*. From Algorithm 1, nonfaulty agents must decide in round *t* + 4 and faulty agents decide before round *t* + 4.

*Validity*. Since no inconsistency is detected, all agents make decisions different from ⊥. For agent *i*, if *i* decides a value decision_*i*_, decision_*i*_ must be the initial preference of an agent in decision set *D*. Since *D* depends on *HS*_*i*_^*m*^*∗*^^, it must have *D*⊆*N*. Therefore, decision_*i*_ satisfies the validity property. If *i* decides ||, *i* has no decision and || does not affect the final consensus outcome. Thus, it also conforms to the validity.

*Uniform Agreement*. We prove this from the following cases:

(i) *Case 1*. Agents *i* and *j* ∈ *Nf*^*t*+4^. By Corollary 3, there must be a decision round *m*^*∗*^ in *t* + 3 rounds. And by Lemma 1, we have *HS*_*i*_^1∼*m*^*∗*^+1^ = *HS*_*j*_^1∼*m*^*∗*^+1^. Then *D*_*i*_ = *D*_*j*_ = *D*^*∗*^. Since the pieces of preferences and proposals of all the agents in *D*^*∗*^ must be saved by at least 2 good agents (Lemma 6), all good agents and some risk agents can restore all initial values and proposals of the agents in *D*^*∗*^ in round *t* + 3. We denote these agents by *Nf*_1_^*t*+4^ and *Nf*_2_^*t*+4^ = *Nf*^*t*+4^*Nf*_1_^*t*+4^.Thus, all agents in *Nf*_1_^*t*+4^ have the same set *C* and give a unified *consensus* set containing one value. That is, if agent *u* and *v* ∈ *Nf*_1_^*t*+4^, then there must be consensus_*u*_ = consensus_*v*_ = {cons} and |consensus_*u*_| = |consensus_*v*_| = 1 in round *t* + 3. And if agent *w* ∈ *Nf*_2_^*t*+4^, consensus_*w*_ = ∅ in round *t* + 3.
(ii) *Case 2*. We analyze the agents in *F*^*t*+4^. It is easy to see that *F*^*t*+4^ = *F*^*t*+2^ ∪ *F*^Δ*t*+3^ ∪ *F*^Δ*t*+4^.
  1. *Case 2.1*. *i* ∈ *F*^*t*+2^. *i* must decide || at the latest in the receive phase of round *t* + 3.
  2. *Case 2.2*. *i* ∈ *F*^Δ*t*+3^. Suppose that *F*_1_^Δ*t*+3^ denotes the set of faulty agents that can receive the messages from some good agents in round *t* + 3 and send messages in round *t* + 4. Then *F*_2_^Δ*t*+3^ = *F*^Δ*t*+3^\*F*_1_^Δ*t*+3^.It is easy to see that the agents in *F*_2_^Δ*t*+3^ definitely do not send messages in round *t* + 4 and they must decide || in round *t* + 3. If *i* ∈ *F*_1_^Δ*t*+3^, by Lemma 11, *D*_*i*_ must be the same as *D*^*∗*^ of good agents in case 1. Thus, if *i* can restore all initial preferences and proposals in *D*_*i*_, it must have consensus_*i*_ = {cons} and |consensus_*i*_| = 1 in round *t* + 3. Otherwise, consensus_*i*_ = ∅ in round *t* + 3.
  3. *Case 2.3*. *i* ∈ *F*^Δ*t*+4^. Since *i* is a nonfaulty agent in round *t* + 3, consensus_*i*_ has the same two possible states in round *t* + 3 as in case 1. Suppose *F*^Δ*t*+4^ = *F*_1_^Δ*t*+4^ ∪ *F*_2_^Δ*t*+4^. *F*_1_^Δ*t*+4^ denotes the set of faulty agents that cannot detect faulty by itself in the receive phase of round *t* + 4. *F*_2_^Δ*t*+4^ denotes the set of faulty agents that can detect that they become faulty agents in the receive phase of round *t* + 4. Therefore, the agents in *F*_1_^Δ*t*+4^ decide *cons* by consensus_*i*_ and the agents in *F*_2_^Δ*t*+4^ decide || in round *t* + 4.

In summary, consensus set only has two types in round *t*+3 and round *t*+4: {cons} and ∅. The agents in *Nf*_1_^*t*+4^ ∪ *Nf*_2_^*t*+4^ ∪ *F*_1_^Δ*t*+4^ decide cons in round *t*+4, the agents in *F*^*t*+2^ ∪ *F*^Δ*t*+3^ decide || before round *t*+4, and the agents in *F*_2_^Δ*t*+4^ decide || in round *t*+4. Thus, uniform agreement holds.

To achieve the Nash equilibrium, we make some appropriate assumptions about initial preferences and failure patterns. Failure pattern represents a set of failures that occur during an execution of the consensus protocol [12]. Specifically, we assume that initial preferences and failure patterns are blind.

**Definition 4 .**

The blind initial values mean that each agent cannot guess the preferences of other agents and the probability of its own preference becoming the consensus cannot be improved by trusting others.

By Definition 4, we can get that if an agent wants to improve its own utility, it can only rely on itself, for example, increasing the probability of entering the decision set and reducing the number of agents in the decision set and so on.

**Definition 5 .**

The blind failure patterns mean that before *t* faulty agents appear, an agent cannot guess the link states in the following rounds. Then we have that

- If agent *i* does not know the link states of round *m* in round *r* and *j* is a nonfaulty agent in round *r*, then*P*(*i* ∈ *F*^Δ*m*^*|*link_*ij*_ is faulty) = *P*(*j* ∈ *F*^Δ*m*^*|*link_*ij*_ is faulty) ≤ *α*.
- For round *m*_1_ and *m*_2_, if *m*_1_ < *m*_2_ and *i* does not know the link states of round *m*_2_, then *P*(*v*_*i*_becomesconsensus*|m*_1_*isthe*decisionround) = *P*(*v*_*i*_becomesconsensus*|m*_2_ is the decision round).
- For *t* + 1 rounds, if the link states of each round in *t* + 1 rounds are unknown to agent *i*, then for a round *r* in *t* + 1 rounds,*P*(*r* is a clean round) ≥ 1/(*t* + 1).

**Theorem 3 .**

If *n* > 2*t*+1, at most *t* agents have omission failures at the same time, agents prefer consensus, and failure patterns and initial preferences are blind, then ${\overset{⟶}{\sigma }}^{\text{CONSENSUS}}$ is a Nash equilibrium.

**Proof**

To prove Nash equilibrium, we need to show that it is impossible for each agent *i* ∈ *N* to increase its utility *u*_*i*_ with all possible deviations *σ*_*i*_. That is, proving that for each agent *i*, there must be

(1) $$\begin{array}{c} u_{i}\left(\sigma_{i}^{\text{CONSENSUS}},\sigma_{-i}^{\text{CONSENSUS}}\right)\geq u_{i}\left(\sigma_{i},\sigma_{-i}^{\text{CONSENSUS}}\right). \end{array}$$

We use the same deduction method as in [12]. Consider all the ways that *i* can deviate from the protocol to affect the outcome as follows:

1. *i* generates a different value *v*_*i*_′ ≠ *v*_*i*_ (or proposal_*i*_′ ≠ proposal_*i*_) and sends *q*_*i*_′(*j*) (or *b*_*i*_′(*j*)) to some agents *j* ≠ *i*.
2. *q*_*i*_(*j*) (or *b*_*i*_(*j*)) sent by *i* cannot restore *v*_*i*_ (or proposal_*i*_).
3. *i* does not choose random_*i*_ or *X*-random_*i*_ or proposal_*i*_ appropriately, such as not randomly.
4. *i* sends an incorrectly formatted message to *j* ≠ *i* in round *m*.
5. If |lost_*i*_| > *t* in round *m*, *i* does not decide ||, but continues to execute the following protocol.
6. *i* lies about the state of *l*_*kp*_ in round *m*; that is, in round *m*, *i* sends a state of *l*_*kp*_ which is different from *NS*_*i*_^*m*−1^[*l*_*kp*_].
7. *i* sends an incorrect random or *X*-random of *l*_*kp*_ to *j* in round *m*.
8. *i* sends an incorrect *q*_*l*_(*i*) (or *b*_*l*_(*i*)) to *j* ≠ *l* different from the *q*_*l*_(*i*) (or *b*_*l*_(*i*)) that *i* has received from *l* in round 1.
9. *i* sends an incorrect consensus_*i*_ to *j* ≠ *i* in round *t* + 4.
10. *i* pretends to crash in round *m*.

We consider these deviations one by one and prove that *i* does not gain by any of deviations. That is, equation (1) holds if *i* deviates from the protocol by these deviations on the list above.

(i) *Type 1*. (i) If *i* sends *q*_*i*_′(*j*) to some agents, then either an inconsistency is detected because of secret restoring error, or *i* does not gain. Specifically, if *i* is the agent whose value is chosen, then *i* is worse off if it lies than it does not, since some agents cannot restore *v*_*i*_, but they can restore it when following the protocol. Then if *i* is not the agent whose preference is chosen, then it does not affect the outcome. (ii) *i* sends *b*_*i*_′(*j*). Then an inconsistency is detected if restoring polynomial error or generating different consensus values in the system. And if no inconsistency is detected, then either all agents that receive *b*_*i*_ or *b*_*i*_′ are faulty or both *b*_*i*_ and *b*_*i*_′ do not affect the final outcome. Since changing the proposal cannot increase *i*'s utility, *i* does not gain. Therefore, both (i) and (ii), *i* does at least as well if *i* uses the strategy *σ*_*i*_^CONSENSUS^, as it deviates from the protocol according to type 1. So, equation (1) holds.
(ii) *Type 2*. It is easy to see that either an inconsistency is detected or no benefit because there is no increase in the probability that *v*_*i*_ becomes the consensus. Thus, equation (1) holds.
(iii) *Type 3*. (i) Since other agents follow the protocol, it does not affect the final outcome because the two kinds of random numbers are only used for verification. (ii) Since *i* does not know the proposals of other agents in round 1, then using different proposals cannot improve the probability that *v*_*i*_ becomes the consensus. Thus, equation (1) holds.
(iv) *Type 4*. If *i* sends an incorrectly formatted message to *j*, then either an inconsistency is detected by *j* or it does not affect the outcome since *j* omits to receive messages from *i* in round *m*. Thus, *i* does not gain, so equation (1) holds.
(v) *Type 5*. Since |lost_*i*_| > *t*, *i* does not receive messages from at least *t* + 1 agents in round *m*, that is, *i* has receiving omission failures with at least two good agents.
  (1) *Case 1*. *i* does not guess message random numbers in round *m* + 1. Then by Claim 1, an inconsistency is detected.
  (2) *Case 2*. *i* guesses the message random number in round *m* + 1 and has correct links with the remaining *n* − *t* − 2 agents, and these agents are all nonfaulty agents. Then by the Claim 1, *i* can successfully send messages in round *m* + 1 iff *i* can guess a message random number random_*j*_^*m*^ from a nonfaulty agent *j* and *i* has no sending omission failures with *j*. That is, the random guessing does not change the detection result of the state of *i* by other agents in round *m*. Clearly the probability that *i* can guess a random number is 1/*n*.
    (a) *Case 2.1*. If *i* guesses some random numbers from agents *j* and has sending omission failures with each agent *j*, then it does not affect the outcome even if the random numbers are correct guesses.
    (b) *Case 2.2*. Suppose that *i* guesses only one random number from the good agent *j* in a round and *i* does not have sending omission failures with *j*. If *i* only guesses the message random number in round *m* + 1, then we have that
      (2) $$\begin{array}{c} u_{i}\left(\sigma_{i},\sigma_{-i}^{\text{CONSENSUS}}\right)\leq \frac{1}{n}P\left(\text{the decision round is in }m+1\text{ rounds}\right)\frac{1}{\left|D\right|}\beta_{0}+\gamma_{1}\beta_{1}+\gamma_{2}\beta_{2}. \end{array}$$
    Thus,
      (3) $$\begin{array}{c} u_{i}\left(\sigma_{i},\sigma_{-i}^{\text{CONSENSUS}}\right)\leq \frac{1}{n}\times \frac{1}{n-t+1}\beta_{0}+\gamma_{1}\beta_{1}+\gamma_{2}\beta_{2}, \end{array}$$
    which means that *i* only guesses one random number in round *m* + 1 and then *i* must be in decision set *D*(|*D*| ≥ |*G*| = *n* − *t*). Since *i* ∈ *D*, there are at least *n* − *t* + 1 agents in *D*. It is easy to see that if *i* guesses random numbers in multiple rounds, the utility must be less than (3). If *i* follows the protocol, then *i* must decide || in round *m*. Since *i* has receiving omission failures in round *m*, the link states after round *m* − 1 must be unknown to *i*. Thus, by Definition 1 and the assumptions about omission failures, we can get that
      (4) $$\begin{array}{c} P\left(\text{round }m-1\text{or }m\text{ is a clean round}\right)\geq \frac{1}{t+1}. \end{array}$$
    Then
      (5) $$\begin{array}{c} u_{i}\left(\sigma^{\text{CONSENSUS}}\right)\geq \frac{1}{t+1}\times \frac{1}{{\left|D\right|}_{m-2}}\beta_{0}+\gamma^{c}\beta_{1}. \end{array}$$
    Since *n* > 2*t* + 1, (1) must hold.
    (c) *Case 2.3*. If *i* guesses more than one random numbers in round *m* + 1, then the utility of *i* must be less than (3). And *i* does at least well by following the protocol as the deviation because the guessing work does not affect the state of *i* in round *m*. Specifically, either if *i* is a nonfaulty agent detected by other agents, then (5) holds, or if *i* is a faulty agent, then it does not affect the outcome even if *i* guesses the random correctly. Thus, (1) holds.
  (3) *Case 3*. If *i* has sending omission failures with the remaining *n* − *t* − 2 agents, then either no benefit since *i* is faulty in round *m* detected by other agents, or *i* does not gain if *i* is nonfaulty because the utility of deviating from the protocol must be less than (3).
In summary, either an inconsistency is detected by Claim 1 if *i* does not guess the message random numbers, or no benefit from guessing the message random numbers. Thus, yet again, (1) holds.
(vi) *Type 6*. By the proof of Type 5, it is easy to see that *i* must be a nonfaulty agent detected by *i* itself in round *m* − 1. Since there is more than one state of a link, we partition this deviation into eight cases and show that *i* does at least well by using *σ*_*i*_^CONSENSUS^ as it deviates from the protocol by these eight deviations.
  (1) *Case 1*. *k* or *p* = *i*, such as *k* = *i*, and Type(*NS*_*i*_^*m*−1^[*l*_*kp*_^*r*^]) = *Msg*-*R* where *r* ≤ *m* − 1, and *i* pretends Type(State_*i*_[*l*_*kp*_^*r*^]) = *Msg*-*X* in round *m*.
    (a) *Case 1.1*. *r* < *m* − 1. Then State_*i*_[*l*_*kp*_^*r*^] must be sent in round *r* + 1 in order to enable message chain mechanism to succeed. Since *r* + 1 < *m*, an inconsistency must be detected in round *m*.
    (b) *Case 1.2*. *r* = *m* − 1 and *r* is the decision round *m*^*∗*^. Since *i* does not know the link states of round *m* − 1 in the sending phase of round *m*, by Definition 1, if *p* is a nonfaulty agent in round *m*, then *P*(*i* ∈ *F*^Δ*m*−1^*|i* pretends link_*ip*_is faulty) = *P*(*p* ∈ *F*^Δ*m*−1^*|i* pretends link_*ip*_ is faulty) ≤ *α*. Suppose that the decision set in round *r* is *D* when following the protocol. We can see that |*D*| ≥ 3. (i) If all agents become faulty due to the deviation of *i*, then there is no consensus in the system and *i* does not gain. (ii) If the deviation does not cause no solution and *p* is a nonfaulty agent in round *m* − 1, then we have that
      (6) $$\begin{array}{c} u_{i}\left(\sigma_{i},\sigma_{-i}^{\text{CONSENSUS}}\right)\leq \alpha \left(1-\alpha \right)\frac{1}{\left|D\right|-1}\beta_{0}+\alpha \left(1-\alpha \right)\frac{\left|D\right|-2}{\left|D\right|-1}\beta_{1}+{\left(1-\alpha \right)}^{2}\frac{1}{\left|D\right|}\beta_{0}+{\left(1-\alpha \right)}^{2}\frac{\left|D\right|-1}{\left|D\right|}\beta_{1}+\alpha \beta_{1}. \end{array}$$
    That is,
      (7) $$\begin{array}{c} u_{i}\left(\sigma_{i},\sigma_{-i}^{\text{CONSENSUS}}\right)\leq \frac{\left(1-\alpha \right)\left(\left|D\right|-1+\alpha \right)}{\left(\left|D\right|-1\right)\left|D\right|}\beta_{0}+\left[\alpha \left(1-\alpha \right)\frac{\left|D\right|-2}{\left|D\right|-1}+{\left(1-\alpha \right)}^{2}\frac{\left|D\right|-1}{\left|D\right|}+\alpha \right]\beta_{1}. \end{array}$$
    It is easy to get that
      (8) $$\begin{array}{c} u_{i}\left(\sigma^{\text{CONSENSUS}}\right)=\frac{1}{\left|D\right|}\beta_{0}+\frac{\left|D\right|-1}{\left|D\right|}\beta_{1}. \end{array}$$
    If *α* = 0, then (1 − *α*)(|*D*| − 1 + *α*) in (7) takes its supremum |*D*| − 1. Hence, there must be equation (1). (iii) If *p* is a faulty agent in round *m* − 1, then the deviation does not affect the outcome. Thus, cases (i),(ii), and (iii) hold equation (1).
    (c) *Case 1.3*. *r* = *m* − 1 and *r* = *m*^*∗*^ + 1. Since the link states of next reliable round are unknown to *i*, either there is no solution since all agents become faulty; the deviation of *i* does not affect the final outcome; or by Definition 1, *i* does at least well by using *σ*_*i*_^CONSENSUS^ as it deviates from the protocol because the probability that *v*_*i*_ becomes consensus does not increase. Thus, equation (1) holds.
    (d) *Case 1.4*. *r* = *m* − 1 and *r* ≠ *m*^*∗*^ and *r* ≠ *m*^*∗*^ + 1. Either there is no solution since all agents become faulty or there is no benefit because it does not affect the decision round.
  In summary, all cases in case 1 cannot make *i* gain.
  (2) *Case 2*. *k* or *p* = *i*, such as *k* = *i*, and Type(*NS*_*i*_^*m*−1^[*l*_*kp*_^*r*^]) = *Msg*-*X* where *r* ≤ *m* − 1, and *i* pretends Type(State_*i*_[*l*_*kp*_^*r*^]) = *Msg*-*R* in round *m*. If r < m-1, i does not gain, which is the same as that in case 1.1. If *r* ≤ *m* −1, *i* does not gain, which is the same as that in case 1.1. If *r* = *m* − 1, since *i* is nonfaulty in round *m* − 1 and *i* does not know the link states of round *m* − 1, then by Definition 1, there is no benefit in guessing the message random number with the probability 1/*n*. So equation (1) holds.
  (3) *Case 3*.*k* and *p* ≠ *i*, and Type(*NS*_*i*_^*m*−1^[*l*_*kp*_^*r*^]) = *Msg*-*R* or *Msg* − *O*, and *i* pretends Type(State[*l*_*kp*_^*r*^]) = *Msg*-*X* in round *m*. Suppose *i* lies about the detection result State_*k*_[*l*_*kp*_^*r*^] of *k*. (i) If *k* is faulty in round *r*, it does not affect the final outcome even if no inconsistency is detected. (ii) If *k* is nonfaulty in round *r*, then *k* must send the faulty random numbers *X*-random_*k*_^*r*^to at least two good agents (Lemma 6). And *i* guesses a faulty random number with the probability 1/2. If it does not affect the states of *k* and *p*, then *i* does not gain even if guessing the random correctly. Otherwise, if the decision round is changed, then
    (9) $$\begin{array}{c} u_{i}\left(\sigma_{i},\sigma_{-i}^{\text{CONSENSUS}}\right)\leq \frac{1}{2}\times \frac{1}{n-t}\beta_{0}+\frac{1}{2}\times \frac{n-t-1}{n-t}\beta_{1}+\frac{1}{2}\beta_{2}. \end{array}$$
  And since *i* is nonfaulty in round *m* − 1, then
    (10) $$\begin{array}{c} u_{i}\left(\sigma^{\text{CONSENSUS}}\right)\geq \frac{1}{n}\beta_{0}+\frac{n-1}{n}\beta_{1}. \end{array}$$
  By the definition of *n* > 2*t* + 1, equation (1) holds. So equation (1) holds both in (i) and (ii).
  (4) *Case 4*. *k* and *p* ≠ *i*, and Type(*NS*_*i*_^*m*−1^[*l*_*kp*_^*r*^]) = *Msg*-*X* or *Msg*-*O*, and *i* pretends Type(State[*l*_*kp*_^*r*^]) = *Msg*-*R* in round *m*. We also suppose that *i* lies about the detection result State[*l*_*kp*_^*r*^] of *k*. (i) If Type(*NS*_*i*_^*m*−1^[*l*_*kp*_^*r*^]) = *Msg*-*O*, then it does not affect the final outcome even if *i* guesses the random correctly, since *Msg*-*R* and *Msg*-*O* have the same meaning when computing the state of an agent. (ii) If Type(*NS*_*i*_^*m*−1^[*l*_*kp*_^*r*^]) = *Msg*-*X*, then either an inconsistency is detected by message random number verification or link state conflict; it does not affect the outcome if the states of *k* and *p* are unchanged after deviating; or it makes round *r* a clean round. Then if there is already a decision round *r*^*∗*^ and *r*^*∗*^ ≤ *r* − 1, it does not affect the outcome because we need the first reliable round finally. And if *r*^*∗*^ > *r* − 1, then the utility of *i* decreases because decision round is advanced compared to following the protocol. If there is no decision round, by Definition 1, *i* does at least well by using *σ*_*i*_^CONSENSUS^ as it deviates from the protocol. Hence, equation (1) holds again.
  (5) *Case 5*. *k* or *p* = *i*, and Type(*NS*_*i*_^*m*−1^[*l*_*kp*_^*r*^]) = *Msg*-*R* or *Msg*-*X* where *r* ≤ *m* − 1, and *i*pretendsType(State_*i*_[*l*_*kp*_^*r*^]) = *Msg* − *O* in round *m*. By Claim 1, an inconsistency is detected. Thus, equation (1) holds.
  (6) *Case 6*. *k* and *p* ≠ *i*, and Type(*NS*_*i*_^*m*−1^[*l*_*kp*_^*r*^]) = *Msg* − *X* or *Msg*-*R*, and *i* pretends Type(State[*l*_*kp*_^*r*^]) = *Msg*-*O* in round *m*. Since *Msg* − *R* and *Msg*-*O* have the same meaning when computing the state of an agent, there is no benefit, which is the same as that in case 4.
  (7) *Case 7*. *k* and *p* ≠ *i*, and Type(*NS*_*i*_^*m*−1^[*l*_*kp*_^*r*^]) = *X*(*r*), and *i* pretends Type(State[*l*_*kp*_^*r*^]) = *X*(*r* − 1) in round *m*. We can turn this case into case 3 because the type of *NS*_*i*_^*m*−1^[*l*_*kp*_^*r*−1^] must be *Msg* − *R*. So it has the same result as that in case 3. Thus, yet again, equation (1) holds.
  (8) *Case 8*. *k* or *p* = *i*, such as *k* = *i*, and Type(*NS*_*i*_^*m*−1^[*l*_*kp*_^*r*^]) = *Msg*-*R* where *r* ≤ *m* − 3, and *i* receivesType(State_*p*_[*l*_*kp*_^*r*^]) = *Msg*-*X*, and *i* pretendsType(State_*p*_[*l*_*kp*_^*r*^]) = *Msg*-*O* or *Msg*-*R* in round *m*. (i) If *p* is a nonfaulty agent in round *r* + 1, then it must sendState_*p*_[*l*_*kp*_^*r*^] to at least two good agents except *i* in round *r* + 1. Thus, all nonfaulty agents must know State_*p*_[*l*_*kp*_^*r*^] in round *m*, so that *i* does not gain. (ii) If *p* is faulty in round *r* + 1, then since *i* is nonfaulty in round *m* − 1, *i* is also a nonfaulty agent in round *r* even if the link between *i* and *p* is faulty. Thus, the results are the same as those in case 4 and case 6. Therefore, equation (1) holds both (i) and (ii).
In summary, in any case, *i*'s utility is at least as high with *σ*_*i*_^CONSENSUS^(*σ*_*i*_, *σ*_−*i*_^CONSENSUS^) as with *u*_*i*_(*σ*_*i*_, *σ*_−*i*_^CONSENSUS^).
(vii) *Type 7*. Since the random numbers are only used in inconsistency detection, either *j* detects an inconsistency and decides ⊥ or it does not affect the final outcome if no inconsistency is detected. Thus, equation (1) holds.
(viii) *Type 8*. It is easy to see that either an inconsistency is detected due to consensus difference or restoring secret faulty; it does not affect the outcome if *l* ∉ *D* or *l* is not the agent whose preference is chosen; or *i* does not gain due to the blind initial preferences by Definition 1 and the random agents' proposals.
(ix) *Type 9*. Clearly it does not affect the outcome if *j* decides || in the receiving phase of round *t* + 4 or *j* does not receive the messages from *i*. Otherwise, *j* must receive the messages from at least two good agents in last round. Then *j* detects an inconsistency and decides ⊥. So equation (1) holds.
(x) *Type 10*. We divide this type into two cases to prove.
  (1) *Case 1*. There is no consensus in the system. This case happens when either all agents become faulty due to the deviation, or restoring secret faulty in round *t* + 3 for all good agents because of the missing pieces of*i*. Thus, *i*'s utility with pretending to crash is lower than with following the protocol in case 1.
  (2) *Case 2*. There is a consensus finally. (i) *m* ≤ *m*^*∗*^. Since *i* does not send messages to any agents before decision round,*i* cannot exist in decision set *D*. Thus, *i*'s utility also decreases when pretending the protocol. (ii) *m* > *m*^*∗*^. It does not affect the outcome. Therefore, (1) holds both in cases 1 and 2.

Finally, concluding the proof.

## 5. Conclusion

In this paper, we introduce game theory as an interpretable method for studying the algorithms in multiagent system and provide an algorithm for uniform consensus that is resilient to both omission failures and strategic manipulations. We prove that our uniform consensus is a Nash equilibrium as long as *n* > 2*t*+1, and failure patterns and initial preferences are blind. Additionally, we present the theory of message passing in presence of process omission failures. We argue that our research enriches the theory of fault-tolerant distributed computing and strengthens the interpretable reliability of consensus with omission failures from the perspective of game theory. And our contribution provides a theoretical basis for the combination of distributed computing and strategic manipulations in omission failure environments, which we think is an interesting research area.

In our opinion, there are many interesting open problems and research directions which are not covered in this paper. We list a few here: (a) whether an algorithm for rational uniform consensus exists if coalitions are allowed; (b) the study of rational consensus with more general types of failures, such as Byzantine failures, is important; (c) with the problem setting of this paper, whether the rational consensus exists if we relax the constraint *n* > 2*t*+1; (d) studying the rational consensus in asynchronous system, which seems significantly more complicated; and (e) introducing the assumption of agent bounded rationality may be useful in practical scenarios.

## Figures and Tables

**Algorithm 1:**
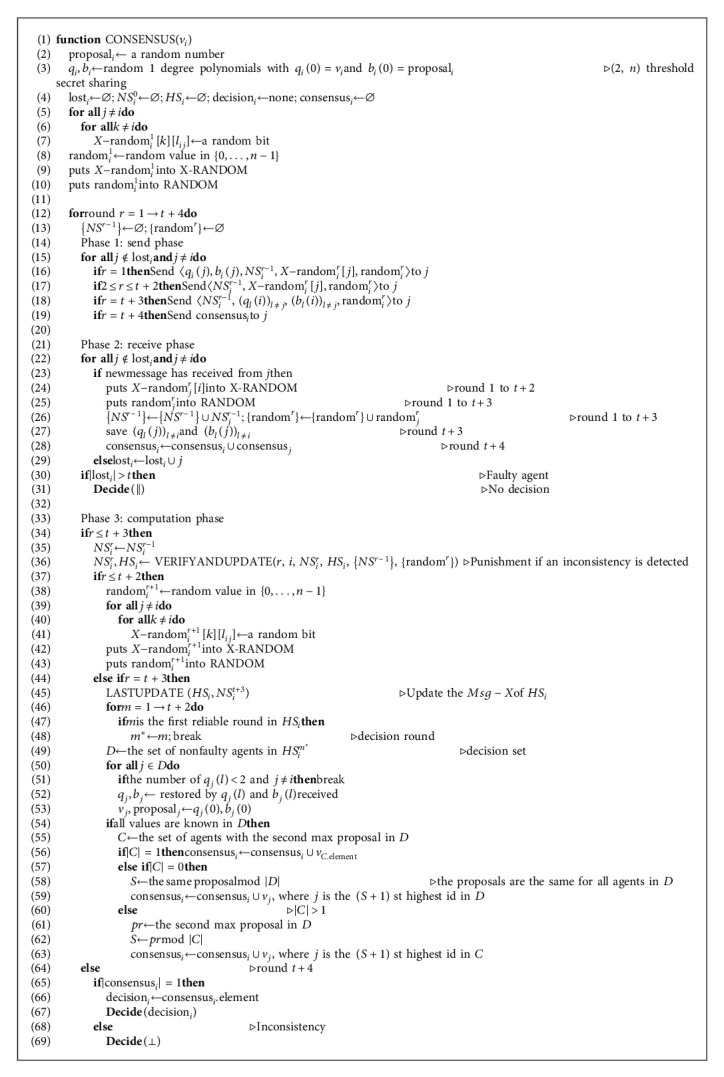
Agent *i*'s uniform consensus protocol with initial value *v*_*i*_(*n* > 2*t*+1).

**Algorithm 2:**
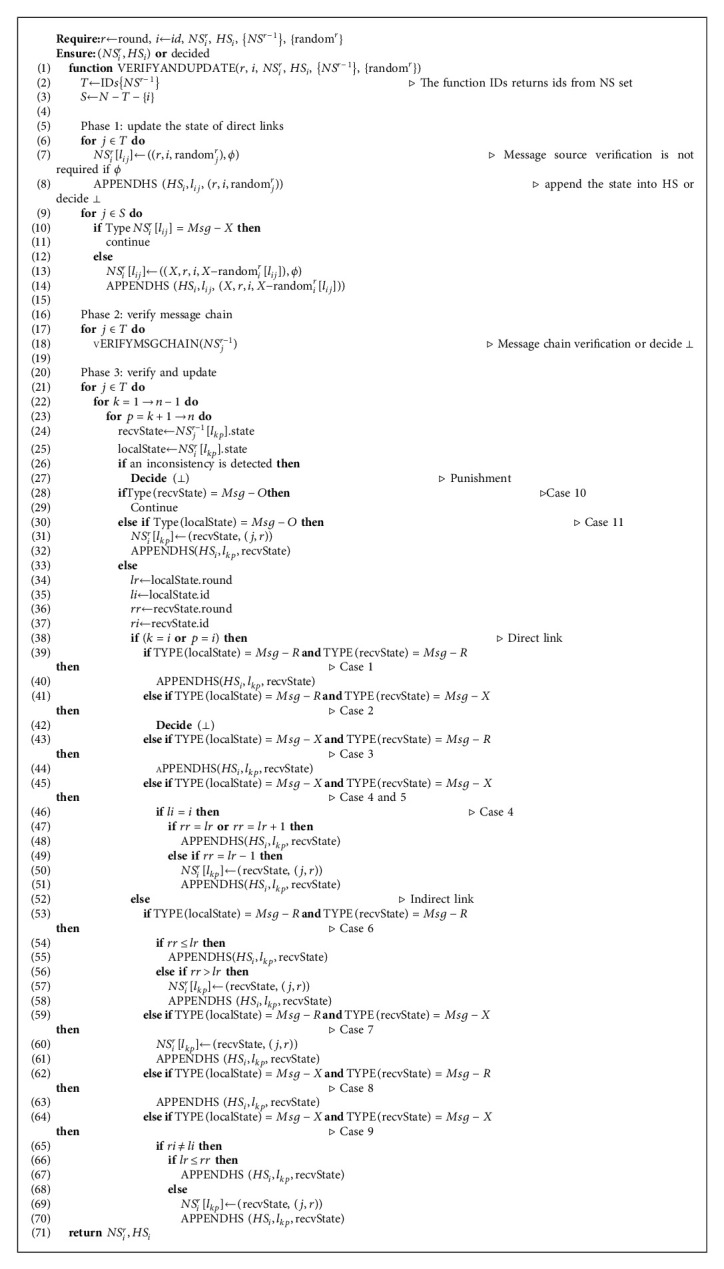
Agent *i* verifies and updates link state in round *r*.

**Table 1:** Notation description.

Variable	Description
link_*ij*_	The link between *i* and *j*
link_*ij*_^*r*^	The link between *i* and *j* in round *r*
*X*-random_*i*_^*r*^	Faulty random number of *i* in round *r*
random_*i*_^*r*^	Message random number for *i* in round *r*
*NS*_*i*_^*r*^	The latest states known to *i* in round *r*
*HS*_*i*_	Historical link states in agent *i*
*v*_*i*_	Initial value of *i*
proposal_*i*_	The number of*i* for computing consensus
*t*_*A*_	The first tuple of *NS*_*i*_^*r*^[*l*_*kp*_]
*t*_*B*_	The second tuple of *NS*_*i*_^*r*^[*l*_*kp*_]
State_*i*_[*l*_*ij*_^*r*^]	Detection result of agent *i* on the state of *l*_*ij*_ in round *r*
*C*_*i*_^*j*^(*m*_1_, *m*_2_)	Agent chain (or message propagation path) from agent *i* to *j*.
*Nf* ^*r*^	Set of nonfaulty agents in round *r*
*F* ^*r*^	Set of faulty agents in round *r*
*F* ^Δ*r*^	Set of faulty agents newly detected in round *r*
*x*_*r*_	The number of risk agents in round *r*

## Data Availability

The data and proof used to support the findings of this study are included within the article.
